# Inter-season training effects on cardiovascular health in American-style football players

**DOI:** 10.1186/s13102-024-00888-4

**Published:** 2024-05-13

**Authors:** Amir Hodzic, Patrick Gendron, Emmanuelle Baron, Amélie Éthier, Odile Bonnefous, Eric Saloux, Paul Milliez, Hervé Normand, François Tournoux

**Affiliations:** 1 Department of Clinical Physiology, Normandie Univ, UNICAEN, CHU de Caen Normandie, Inserm Comete, GIP Cyceron, Caen, 14000 France; 2 Department of Cardiology, Normandie Univ, UNICAEN, CHU de Caen Normandie, Caen, 14000 France; 3 Clinique de Médecine du Sport, University of Montreal, Montreal, Canada; 4 Research Center of the Hospital of the University of Montreal (Centre de Recherche du Centre Hospitalier de L’Université de Montréal), Montreal, Canada; 5 Philips Research, Medical Imaging (Medisys), Suresnes, France; 6 Université Caen Normandie, UMR 1075 COMETE UNICAEN/INSERM, 2 Rue Des Rochambelles, Caen, 14032 France

**Keywords:** Athlete’s heart, American-style Football, Inter-season training, Left ventricular hypertrophy, Left atrial function, 3D echocardiography, Speckle tracking, Intraventricular pressure gradients

## Abstract

**Background:**

Recent studies on American-style football (ASF) athletes raised questions about the impact of training on the cardiovascular phenotype, particularly among linemen players who engage mostly in static exercise during competition and who exhibit concentric cardiac remodeling, often considered maladaptive. We aimed to examine the cardiovascular adaptation to the inter-season mixed-team training program among ASF players.

**Methods:**

A prospective, longitudinal, cohort study was conducted among competitive male ASF players from the University of Montreal before and after an inter-season training, which lasted 7 months. This program includes, for all players, combined dynamic and static exercises. Clinical and echocardiographic examinations were performed at both steps. Left atrial (LA) and ventricular (LV) morphological and functional changes were assessed using a multiparametric echocardiographic approach (2D and 3D-echo, Doppler, and speckle tracking). Two-way ANOVA was performed to analyze the impacts of time and field position (linemen versus non-linemen).

**Results:**

Fifty-nine players (20 linemen and 39 non-linemen) were included. At baseline, linemen had higher blood pressure (65% were prehypertensive and 10% were hypertensive), thicker LV walls, lower LV systolic and diastolic functions, lower LA-reservoir and conduit functions than non-linemen. After training, linemen significantly reduced weight (Δ-3.4%, *P* < 0.001) and systolic blood pressure (Δ-4.5%, *P* < 0.001), whereas non-linemen maintained their weight and significantly increased their systolic (Δ+4.2%, *P* = 0.037) and diastolic (Δ+16%, *P* < 0.001) blood pressure ). Mixed training was associated with significant increases in 2D-LA volume (*P* < 0.001), 3D-LV end-diastolic volume (*P* < 0.001), 3D-LV mass (*P* < 0.001), and an improvement in LV systolic function, independently of the field position. Non-linemen remodeled their LV in a more concentric fashion and showed reductions in LV diastolic and LA reservoir functions.

**Conclusions:**

Our study underscored the influence of field position on cardiovascular adaptation among university-level ASF players, and emphasized the potential of inter-season training to modulate cardiovascular risk factors, particularly among linemen.

## Background

Numerous longitudinal observational studies suggested that American-style football (ASF) participation can lead to early maladaptive cardiovascular remodeling characterized by the development of a concentric left ventricular (LV) hypertrophy associated with reduced LV diastolic function and systolic longitudinal deformation, increased relative arterial stiffening, and metabolic perturbations [1–7]. This cardiovascular maladaptation has been particularly well-documented in linemen players [8]. Due to their positional roles on the field, defensive and offensive linemen experience the lowest running demands during the ASF in-season training and competition. As a result, their training regimen during this period predominantly consists of static exercises to optimize maximal strength and increase fat mass, aiming to enhance performance during competition. In contrast, players in non-linemen positions undergo a more dynamic exercise approach, contributing to heightened aerobic fitness, leading to a distinctive cardiovascular phenotype characterized by eccentric left ventricular hypertrophy, coupled with normal cardiac function [4, 9].

Detraining in players with concentric LV hypertrophy can be used to confirm its benign training-related etiology [10] and distinguish it from maladaptive hypertrophy linked to other factors such as hypertension [7, 11, 12] and obesity [11, 13, 14] which may affect these athletes. Regression of LV hypertrophy by detraining suggests that cardiac adaptation to exercise is dynamic, but data are limited, especially since voluntary detraining usually occurs at the end of an athlete’s career and cardiac imaging in athletes is performed when clinically warranted [15]. Due to the limited off-season duration for elite athletes, it is worth considering a period of the year when physiological responses to exercise may vary. This is particularly true during the inter-season physical preparation phase. Significant differences have been observed in training regimens between the inter-season and in-season competitive periods for the ASF [16]. Inter-season training focuses on enhancing the fitness level of ASF players, including linemen players who typically experience weight gain during both competition and off-season. Furthermore, this period is generally characterized by higher training loads compared to in-season training [16]. However, it remains unclear whether variations in training mode are sufficient to modify the cardiovascular phenotype (including cardiac remodeling and blood pressure), which has been predominantly studied in this population during competition seasons. Exploring the effects of inter-season training on cardiovascular phenotype in ASF players could be valuable in promoting long-term cardiovascular well-being among these athletes, who face an increased risk of cardiovascular complications [17]. 

We hypothesize that modifications in physical training modalities imposed during the ASF inter-season are sufficient to induce distinct cardiovascular responses among university ASF players compared to those reported previously during competition periods. Thus, this study aimed to describe the longitudinal changes in cardiovascular phenotype among ASF linemen and non-linemen players during the inter-season period.

## Methods

### Study design

We carried out a prospective, longitudinal, cohort study to examine the effects of ASF inter-season training on LV and left atrium (LA) responses among competitive male athletes from the University of Montreal, during the 2016 season. Informed consent was obtained from all participants involved in the study. The study was approved by the research ethics committee of the CHUM Research Center (CRCHUM) (IRB#13.253). The eligibility criteria for participation in this study included being a member of the University of Montreal’s ASF team “Les Carabins”, having the ability to attend all study visits, and showing interest in participating in the research. An upcoming first season of football at the University of Montreal was not an exclusion criterion in our study since all the players were either transferred from another university team or engaged in competitive football teams before starting their university studies.

### Protocol

**Fig. 1:**
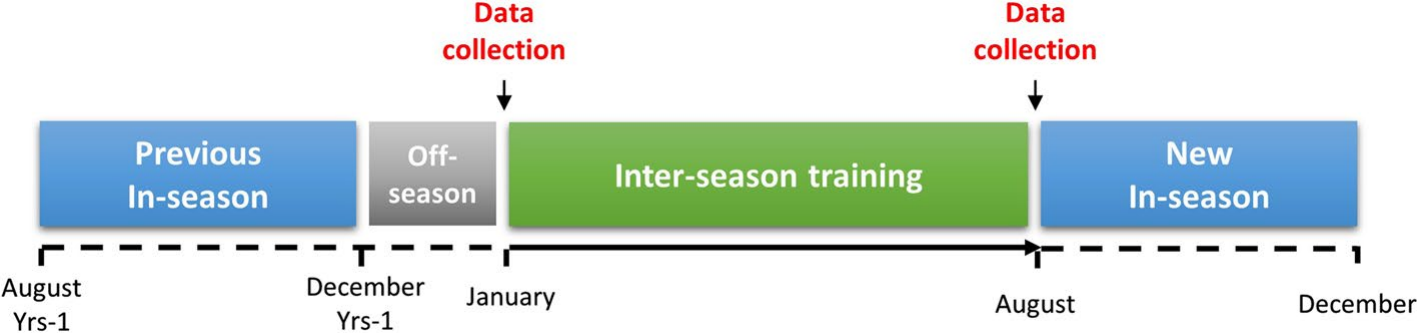
Study flow chart

Data collection for the study occurred during two distinct visits: one conducted immediately before and another at the end of the inter-season training period (Fig. 1). During the initial visit, we obtained declarative information regarding participants’ age, height, personal medical history, chronic treatment, or illicit drug use. Additionally, participants were questioned about the duration of their football participation at the university level and their position on the field, distinguishing between linemen and non-linemen athletes, as previously defined [18]. Measurements including weight, body fat percentage, heart rate, and blood pressure were collected during both visits. Furthermore, cardiac structure and function were analyzed in all participants using transthoracic echocardiography at each visit. Participants were instructed to observe a 24-h exercise-free period, refraining from both endurance and resistance training, before each medical visit.

### Outcome measures

#### Clinical data

At each visit, resting heart rate and blood pressure were measured after 10 min of quiet rest in a supine position. Blood pressure was determined by taking duplicate successive measurements using an automated monitor (OMRON Healthcare, Hoofddorp, The Netherlands) with a properly sized arm cuff on the left arm. The average of the two measurements was then recorded. Body mass index (BMI) was calculated by dividing weight (kg) by the square of height (m), while body surface area (BSA) was calculated using the Mosteller formula [19]. To measure body fat percentage, a tetrapolar bioelectrical impedance analysis was performed using the Tanita Body Composition Analyzer BF-350 (Tanita Corporation, USA). 

#### Echocardiographic analysis

The echocardiographic assessment of LV and LA functions was carried out according to the current guidelines [20], using a commercially available echocardiographic system (EPIQ 7 equipped with an X5-1 xMATRIX-array transducer, Philips). Participants were scanned at the pre-inter-season medical assessment and within one week of completing the inter-season training program. All data were stored digitally, and offline data analysis was performed by a single operator blinded to the study time point (TOMTEC-Arena TTA2, TOMTEC Imaging Systems GMBH, Germany). LV dimensions were assessed from M-mode via the parasternal long-axis view. LV 3D volumes, ejection fraction (EF), and mass were obtained using the TOMTEC 4D LV analysis software. LV global longitudinal strain (GLS) was based on 3D speckle tracking. The geometric relationship between LV 3D end-diastolic volume (EDV) and LV wall thickness was assessed using the Remodeling Index as previously described [21] as $\frac{\sqrt[3]{LV EDV}}{t}$, where t represents in the present paper the average of LV basal wall thickness calculated as follows: [interventricular septal thickness (mm) + posterior wall thickness (mm)]/2.

LV diastolic function was analyzed by Doppler indices. Peak early (E) and late (A) transmitral velocities were measured by pulsed-wave Doppler. Peak mitral lateral annulus early (e’) and late (a’) velocities were obtained by tissue Doppler imaging. Color Doppler M-mode was recorded along the LV base-to-apex axis with the cursor parallel to the mitral inflow in the apical four-chamber view to assess noninvasively the peak of the diastolic intraventricular pressure gradient (DIVPG) [22], a marker of LV diastolic suction [23]. The method used for color Doppler M-mode image processing and DIVPG calculation has been previously described by our team [24]. All Doppler parameters were obtained as the average value of three consecutive cardiac cycles during end-expiratory apnea.

LA volume was estimated by the biplane method of disks at end-systole from apical 4-chamber and 2-chamber views. 2D-speckle tracking measurements of LA phasic strains were performed according to current practice recommendations [25]. The LA reservoir, conduit, and contractile strains were calculated with the first reference frames at the onset of the QRS wave of the surface ECG as recommended [25]. Values were obtained from a single apical 4-chamber view. LA conduit and contractile strains are reported in absolute values. LA stiffness was estimated using the E/e’ and LA reservoir strain ratio, as previously applied for the athlete’s heart [26].

### The inter-season team-training program

The inter-season training program of the University of Montreal ASF team was previously described by our team [27]. Briefly, the training program in this study commenced immediately following the medical visit in late January 2016 and lasted seven months until August 2016. During this period, the participants engaged in team practice sessions under the guidance of professional coaches. The initial training period which lasted 12 weeks focused on anaerobic hypertrophy strengthening exercises that alternated between upper and lower body muscle groups. These exercises were performed for one hour per session, four times per week, and included 8–12 repetitions at an intensity of 70–80% of one repetition maximum (1-RM). A variety of closed kinetic chain exercises, such as squats, lunges, deadlifts, bench press, curls, chin-ups, and core strengthening exercises were used. In addition, the participants engaged in progressive moderate aerobic to high-intensity anaerobic pace running training for three hours per session, three times per week. During the subsequent phase, which commenced in April, the strengthening exercises were modified to anaerobic maximum strengthening. The participants were required to lift loads greater than 80% of 1-RM, and the repetitions were reduced to five sets of six repetitions of the same exercises used in the previous phase. The running program was also altered to include high-intensity short-distance sprints and cuttings. This phase lasted for twelve weeks with one hour and thirty minutes per session, four times per week. In mid-June, the training program focused on power-strengthening exercises. These included a mix of anaerobic powerlifting and plyometric exercises, such as clean, snatches, jumps, and explosive bench press exercises, and were performed at the same weekly frequency as the previous phase. The running program remained the same. During the entire inter-season period, linemen were advised to replace one running session with low-intensity aerobic training, mobility, and football technique training. They were also instructed to monitor their diet and lose any excess weight gained during the low training season, from November to January. Finally, in early August, the participants underwent a medical reassessment before the start of the competition season.

### Statistical analysis

Statistical analysis was performed with MedCalc Statistical Software (version 13.2.0, MedCalc Software bvba, Belgium). A *P*-value of < 0.05 was considered significant. All eligible volunteers from the team were included in the analysis without sample size calculation. Categorical variables are presented as proportions and continuous variables as mean ± standard deviation. The normality of distribution was verified using the Shapiro–Wilk test. Paired data were compared using Student’s paired *t*-test or McNemar’s exact test while unpaired data were compared using the Student’s independent sample *t*-test or Fisher’s exact test. Two-way analysis of variance (ANOVA) for repeated measures assessed before and after the inter-season with the Tukey–Kramer posthoc test, was used to test the significance of the observed changes with time and to look for potential group interaction (linemen versus non-linemen). Prior to the ANOVA, Levene’s test for equality of variances was performed.

## Results

### Baseline characteristics

Of the 72 male participants who consented to the study, 59 (20 linemen and 39 non-linemen) completed the full inter-season program and thus were analysed at both study time points. A full LV 3D analysis was not feasible in 5 linemen due to insufficient image quality (insufficient echogenicity for 4 participants, and respiratory artifacts for the other one). The mean age was 21.9 ± 1.4 yrs. There was no age difference between linemen and non-linemen (Table 1). Prior to the start of the inter-season training linemen had significantly higher weight (128.4 ± 16.9 kg *vs.* 88.7 ± 9.8 kg, *P* <  0.001), BMI (36.1 ± 4.1 kg/m^2^ *v*s. 27.1 ± 3 kg/m^2^, *P*  < 0.001), and body fat percentage (26.2 ± 6.4% *vs.* 15.3 ± 4.7%, *P* < 0.001) than non-linemen. Similarly, systolic and diastolic blood pressure (SBP, DBP) at pre-training were significantly higher in linemen than in non-linemen (Table 1). Although none of the participants reported a personal history of hypertension or medication, 5% of the participants had a SBP ≥ 140 mmHg at the time of the initial assessment (Table 1). In terms of cardiac analysis, at baseline, linemen demonstrated signs of a more LV concentric remodeling phenotype with functional modifications in comparison with their non-linemen counterparts: they had significantly smaller BSA indexed LV end-diastolic diameter (LVEDD), greater wall thickness, lower Remodeling Index, lower LV EF, lower GLS, lower LV suction (peak DIVPG) and lower longitudinal relaxation (peak e’) (Table 2). The LA volume was larger in linemen but this difference was no longer significant when indexed to BSA (Table 3). However, LA function (reservoir and conduit strains) was significantly lower in linemen at baseline with an increased LA stiffness index compared to non-linemen (Table 3).

**Table 1:** Clinical data

**Variable**	**All athletes (*n*=59)**			**Non-linemen (*n*=39)**			**Linemen (*n*=20)**			***Interaction*** Time*Group
	***Pre-training***	***Post-training***	***P-value***	***Pre-training***	***Post-training***	***P-value***	***Pre-training***	***Post-training***	***P-value***	
Age, years	21.9±1.4			21.8±1.4			22±1.5			
Prior university ASF seasons	1.6±1.1			1.6±1.1			1.7 ± 1.1			
Height, cm	183.6±7.2			181.2±6.1			188.3±7.1*			
Weight, kg	102.3±22.6	101±20.4	**0.021**	88.7±9.8	89.3±9.7	0.25	128.4±16.9***	123.9±16†††	**0.0003**	**<0.001**
BMI, kg/m^2^	30.1±5.5	29.8±4.8	**0.029**	27.1±3	27.2±2.9	0.24	36.1±4.1***	34.8±3.8†††	**0.0004**	**<0.001**
BSA, m^2^	2.28±0.28	2.26±0.25	**0.033**	2.1±0.1	2.1±0.1	0.27	2.6±0.2***	2.5±0.2†††	**0.0002**	**<0.001**
Body fat, %	19±7.4	18.2±6.6	0.068	15.3±4.7	15.2±4	0.85	26.2±6.4***	24.2±6.7†††	**0.038**	**0.018**
Heart rate, beats/min	58.9±7.9	60.2±8.1	0.18	57.8±7.8	60.4±8.4	**0.011**	60.4±8.2	59.7±7.7	0.72	0.07
Systolic BP, mmHg	121.6±10.6	122.4±9.4	0.68	118.2±8.8	122.5±9.5	**0.037**	128.2±11.1***	121.8±9.5	**0.038**	**0.003**
Diastolic BP, mmHg	67±8.3	73.9±8	**<0.0001**	64.5±7.3	74.1±7	**<0.0001**	72.2±7.8***	73.6±9.8	0.53	**0.001**
Systolic BP <120 mmHg	28 (47%)	21 (36%)	0.25	23 (59%)	12 (31%)	**0.019**	5 (25%)**	9 (45%)	0.29	-
Systolic BP 120-139 mmHg	28 (47%)	36 (61%)	0.2	15 (38%)	25 (64%)	**0.04**	13 (65%)	11 (55%)	0.75	-
Systolic BP ≥140 mmHg	3 (5%)	2 (3%)	0.99	1 (3%)	2 (5%)	0.99	2 (10%)	0	0.5	-

Values are means±SD

*Abbreviations: BMI* body mass index, *BP* blood pressure, *BSA* body surface area

^*^P<0.05 **P<0.01 ***P<0.001 for comparison between non-linemen and linemen measurements at the pre-training visit

^†††^*P*<0.001 for comparison between non-linemen and linemen measurements at the post-training visit

**Table 2:** Left ventricular morphological and functional parameters

**Variables**	**Non-linemen (*n*=39)**			**Linemen (*n*=20, 15 for 3D data)**			***Interaction*** Time*Group
	***Pre-training***	***Post-training***	***P-value***	***Pre-training***	***Post-training***	***P-value***	
***LV morphology***							
LVEDD, mm	52.3±3.7	53.4±3.8	**0.005**	57.3±4.8***	58.8±3.9†††	**0.007**	0.64
LVEDDi, mm/m^2^	24.8±1.8	26±5.3	**0.08**	22.3±2.5***	23.2±2.1†	**0.0003**	0.77
LVESD, mm	38.1±3.2	37.4±3.6	0.14	43.3±4***	42.8±3.8†††	0.37	0.76
LVESDi, mm/m^2^	18.1±1.5	17.7±1.7	0.07	16.8±1.9**	16.9±1.8	0.7	0.17
IVSd, mm	7.9±1.1	8.4±1.1	**0.0005**	8.9±0.7**	9.3±0.9††	**0.008**	0.66
PWTd, mm	7.5±0.9	7.9±0.9	**0.004**	8.6±0.8***	8.8±0.9†††	0.18	0.58
3D-LVEDV, ml	163.2±22.1	171±23.5	**<0.0001**	201.4±35.5***	212.6±35.8†††	**0.0006**	0.4
3D-LVEDVi, ml/m^2^	77.3±9.5	81.1±9.3	**<0.0001**	78.1±12.7	83.9±12.5	**0.0002**	0.18
3D-LVESV, ml	71.6±11.5	72.7±11.7	0.41	96.4±18.4***	96.2±21.5†††	0.97	0.68
3D-LVESVi, ml/m^2^	33.9±4.8	34.3±4.7	0.53	37.2±6*	37.9±7.6†	0.64	0.83
3D-LV mass, g	168.9±19.9	181.6±21	**<0.0001**	205.1±31.2***	220.6±33.9†††	**<0.0001**	0.42
3D-LV mass i, g/m^2^	80.1±8.8	85.8±8.4	**<0.0001**	79.5±10.3	87±11.2	**<0.0001**	0.24
3D-Remodeling Index	7.14±0.84	6.87±0.73	**0.006**	6.51±0.67*	6.39±0.59†	0.1	0.46
***LV function***							
LV ejection fraction, %	56.2±3.6	57.5±3.8	**0.049**	52.2±3.6***	54.9±5†	0.08	0.34
LV stroke volume, ml	91.7±13.4	99.2±15.2	**<0.0001**	105±19.6**	116.3±19.2††	**0.001**	0.19
LV GLS, %	-19.9±2	-20.7±2.2	**0.02**	-18.7±1.7*	-19.5±2.8	0.24	0.89
Transmitral E-wave, cm/s	81.6±13	79.3±11.7	0.18	75±13.9	76.6±12.7	0.55	0.2
Transmitral A-wave, cm/s	42.2±9.6	39.5±8.7	0.08	45.7±12.3	44±10.8	0.59	0.74
E/A ratio	2±0.7	2.1±0.5	0.62	1.7±0.4	1.8±0.6	0.34	0.65
DIVPG, mmHg	-2.93±0.8	-2.85±0.84	0.53	-2.37±0.85*	-2.28±0.91†	0.71	0.85
TDI e’ basal, cm/s	16.7±2.6	15.8±2.8	**0.01**	14.4±2.9*	14.4±2.7	0.99	0.1
TDI a’ basal, cm/s	5.6±1.3	6.1±1.5	**0.02**	6.2±1.3	6.1±1.2	0.85	0.15
E/e’ ratio	5±1.1	5.2±1.1	0.24	5 2±0.9	5.2±0.8	0.96	0.51

Values are means±SD

*Abbreviations: DIVPG* diastolic intraventricular pressure gradient, *GLS* global longitudinal strain, *i* indexed, *IVSd* interventricular septal thickness in diastole, *LV* left ventricle, *LVEDV* LV end-diastolic volume, *LVESV* LV end-systolic volume, *LVEDD LV end-diastole diameter*, *LVESD LV end-systole diameter*, *PWTd* posterior wall thickness in diastole, *TDI* tissue Doppler imaging

^*^P<0.05 **P<0.01 ***P<0.001 for comparison between non-linemen and linemen measurements at the pre-training visit

^†^*P*<0.05 ††*P*<0.01 †††*P*<0.001 for comparison between non-linemen and linemen measurements at the post-training visit

**Table 3:** Left atrial morphological and functional parameters

**Variable**	**Non-linemen (*n*=39)**			**Linemen (*n*=20)**			***Interaction*** Time*Group
	***Pre-training***	***Post-training***	***P-value***	***Pre-training***	***Post-training***	***P-value***	
***LA morphology***							
LAV, ml	59.7±11.8	67.7±12.9	**<0.0001**	70.5±15.8**	77.8±16.2†	**<0.0001**	0.68
LAVi, ml/m^2^	28.3±5.2	32±5.6	**<0.0001**	27.4±6.7	30.9±7.3	**<0.0001**	0.75
***LA function***							
Reservoir strain, %	38.4±5.7	36±7.6	**0.048**	32.3±5.7***	30.8±4.7††	0.23	0.6
Conduit strain, %	28.1±4.6	27.5±5.9	0.47	21.9±5.2***	22.3±4.7††	0.63	0.46
Contractile strain, %	11±3.5	11.1±3.6	0.93	11.5±3.2	11.3±3.9	0.87	0.84
Stiffness index	0.13±0.03	0.15±0.05	**0.011**	0.16±0.04**	0.17±0.04	0.51	0.32

Values are means±SD

*Abbreviations: LA* left atrial, *LAV* left atrial volume, *BSA* body surface area, *i* indexed

^**^P<0.01 ***P<0.001 for comparison between non-linemen and linemen measurements at the pre-training visit

^†^*P*<0.05 ††*P*<0.01 for comparison between non-linemen and linemen measurements at the post-training visit

### Longitudinal effects of inter-season training

**Fig. 2:**
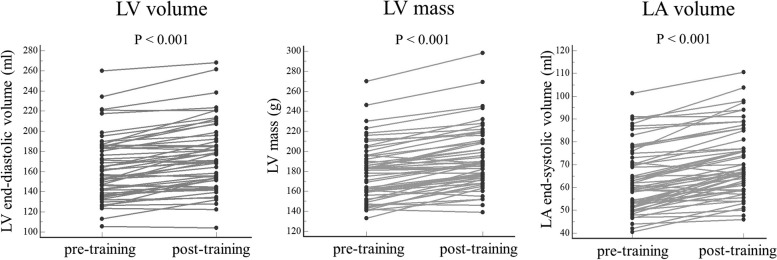
Individual longitudinal changes in left ventricular (LV) and left atrial (LA) structure among ASF athletes

At the end of the inter-season training program, linemen had significant reductions in weight (Δ = -4.5 ± 4.6 kg), BMI (Δ = -1 ± 1.3 kg/m^2^), and body fat percentage (Δ = -2 ± 3.6%), while non-linemen did not experience any significant changes in those parameters (Table 1). Additionally, the arterial response at the end of training significantly differed between the two groups. Only linemen showed a significant reduction in SBP, while non-linemen experienced increases in both SBP and DBP (Table 1). LV size and mass both increased significantly after the training period (Fig. 2), regardless of the field position. Similarly, systolic function assessed by EF, stroke volume, and GLS was also slightly improved at the second visit (Table 2). The Remodeling Index and the longitudinal relaxation parameter peak e’ significantly decreased over time. This reduction was observed only in the non-linemen group of athletes but considering the *p*-value for the interaction factor, there was no confirmed significant group effect (Table 2). LV suction and E/e’ratio did not significantly change during the observed period (Table 2). Finally,at the end of inter-season training, LA volume significantly increased (Fig. 2), irrespective of the field position (Table 3). Only LA reservoir strain and LA stiffness changed over the inter-season training period, with a trend for this change only in non-linemen athletes (Table 3).

## Discussion

Key findings from this study can be outlined as follows: Firstly, our data confirmed that field position significantly influenced the cardiovascular profile of university-level ASF players, with linemen showing particularly notable differences. They exhibited elevated body fat mass, higher blood pressure profiles, a more concentric left ventricular remodeling, and lower diastolic ventricular and atrial functions compared to their counterparts. Secondly, our study explored, for the first time, the cardiovascular dynamics effects of inter-season training, which appeared to be particularly beneficial among linemen. This included a decrease in fat mass and arterial blood pressure, along with the absence of alterations in cardiac functional parameters previously associated with ASF seasons. Overall, these findings enhance our understanding of cardiovascular responses in these athletes, suggesting that inter-season training program could partially reverse the cardiovascular phenotype developed among linemen. 

### Effects of inter-season on body composition and blood pressure

Linemen field positions, both offensive and defensive, are commonly associated with higher BMI compared to non-linemen [8]. Longitudinal observations further established a correlation between ASF participation and weight gain [5, 28]. Our data corroborated these findings, revealing that linemen exhibited significantly higher BMI and fat mass than non-linemen. This difference was evident at the initial evaluation and persisted throughout the follow-up period. However, the inter-season period allowed for a significant reduction in both BMI and fat mass among linemen. These observations are particularly significant considering that obesity and fat mass are strong predictors of cardiovascular diseases in the general population, especially when developing at a young age [29, 30]. Although this association has not been clearly established in the ASF population, the reduction in weight and fat mass during our inter-season program were accompanied by an improvement in arterial blood pressure among linemen. Indeed, 75% of the linemen (versus 41% of non-linemen) were considered as being prehypertensive or hypertensive at the initial visit. These findings are similar to previously reported cohorts of athletes involved in the American National Football League [4, 5, 11, 12]. However, the notable aspect of our study is that the evolution of arterial blood pressure measurements during our follow-up differ from what was typically reported in this athletic population. ASF competitive athletes commonly experience increased arterial blood pressure during seasonal participation [7]. Yet, our data showed that inter-season training was associated with a significant reduction in SBP in linemen (with no change in DBP), while non-linemen increased both SBP and DBP. The increase in the dynamic component of exercise training during the inter-season for linemen, who predominantly engage in intense static activity during the competition ASF season, might explain the beneficial effect noted on blood pressure throughout the inter-season, alongside the weight reduction. Nevertheless, this effect was not apparent among non-linemen, who initially exhibited lower blood pressure profiles and experienced a rise in strength training during the same period.

### Effects of inter-season on cardiac phenotype

Although our baseline examination was performed after a detraining period (around 4 weeks) following the end of the previous ASF season, some signs of the field position-specific LV remodeling patterns previously described in the literature [3–5, 28] were still noticeable. Using the Remodeling Index, we observed a significantly different LV geometric relationship between the EDV and the wall thickness in linemen compared to non-linemen. The pertinence of this index has been previously demonstrated in cohorts of hypertensive patients. [21, 31]. This index was derived from the biophysical model of myocardial wall stress (Laplace law) to detect adverse hypertensive LV remodeling using cardiac magnetic resonance imaging. These observations demonstrated that hypertensive patients including those without concentric LV hypertrophy had a lower Remdeoling Index compared to age and sex-matched controls. Furthermore, this index could identify hypertensive patients at a higher risk of developing structural cardiac abnormalities such as myocardial fibrosis and impaired diastolic function and appeared to be predictive of the occurrence of long-term cardiovascular events [21, 31]. We adapted this concept to 3D echocardiography and found baseline values in the non-linemen participants similar to the normal range established by these authors in a healthy male control population aged between 20–29 years old (mean value of 7.1). Conversely, the same index used in linemen showed values significantly lower and closer to the mean values reported in a healthy male population 10 to 30 years older (mean values between 6.8 to 6.4) [21]. Since ASF participation classically exposes the linemen players to a higher LV pressure overload compared to non-linemen ones, they usually demonstrate greater LV wall thickening to maintain wall stress which can lead to a form of concentric hypertrophy [4, 5]. This structural remodeling can be associated with some functional impairment as objectified in our cohort where linemen at baseline had a slightly but significantly lower LV systolic function, longitudinal relaxation, and suction (a marker of LV untwisting [32]). Besides, non-linemen who experienced a seasonal increase in blood pressure exhibited LV hypertrophy characterized by concomitant reductions in LV peak e’ and Remodeling Index. While seasonal cardiac remodeling in linemen seemed to evolve into an eccentric LV geometry, these participants experienced no increase in LV diastolic parameters. These observations suggest a possible uncoupling of LV morphology and diastolic function and might indicate the early onset of potential disorders in the myocardial diastolic proprieties among linemen. Premature chronic exposure to high blood pressure and obesity, well-documented in linemen players [8], have been previously associated in the general population with evidence of early LV diastolic alterations that could occur independently of LV hypertrophy [33, 34]. Early LV diastolic impairment among linemen was also suggested by our LA functional analysis. LA reservoir and conduit functions are known to be modulated by LV diastolic properties [35]. Our speckle tracking analysis confirmed that although LA morphological remodeling was similar between groups, linemen exhibited much lower LA reservoir and conduit functions compared to their non-linemen counterparts and compared to the mean values reported in trained athletes including strength athletes [36, 37]. LA reservoir was slightly reduced at the end of the inter-season in both groups, which could be related to the training-induced atrial enlargement [38].

### Limitations

Several limitations of this study are noteworthy Firstly, the absence of quantitative data on individual weight and diet management precludes a thorough analysis of the causal relationship between weight and fat mass loss and the observed cardiovascular response. Moreover, we did not examine the potential presence of other risk factors, such as tobacco use, the presence of metabolic syndrome or sleep-disordered breathing, that are known to be relevant in cardiovascular adaptation [8]. Similarly, relying on self-reported information for assessing doping substances, we cannot exclude a potential bias associated with self-declaration accuracy. In addition, the consumption of recreational drugs was not investigated. Finally, we acknowledge the size of the studied cohort was limited, and we did not conduct sample size calculation. However, we are not aware of any other reports of longitudinal evaluation during this inter-season period among ASF team participants.

## Conclusion

Our study aligns with previous research, underscoring concerns regarding the impact of ASF participation on cardiovascular phenotype, especially among lineman-position players. However, our data showed that a single inter-season training program led to significant modifications in cardiovascular risk factors among linemen, including reductions in fat mass and arterial pressure, while non-linemen showed differing effects. Further longitudinal studies are required to delve deeper into the mechanisms underlying the early development of cardiovascular risk factors associated with ASF participation. Additionally, exploring the efficacy of targeted approaches in mitigating these risks and promoting long-term cardiac health among ASF athletes is warranted.

## Data Availability

The data presented in this study are available on request from the corresponding author.

## Abbreviations

A: Peak transmitral A-wave
ASF: American-style football
a’: Peak end-diastolic tissue velocity
BMI: Body mass index
BSA: Body surface area
DBP: Diastolic blood pressure
DIVPG: Diastolic intraventricular pressure gradient
E: Peak transmitral E-wave
EDV: End-diastolic volume
EF: Ejection fraction
e’: Peak early diastolic tissue velocity
GLS: Global longitudinal strain
LA: Left atrium
LV: Left ventricle
LVEDD: Left ventricular end-diastolic diameter
RM: Repetition maximum
SBP: Systolic blood pressure

## References

[1] Baggish AL, Wang F, Weiner RB, Elinoff JM, Tournoux F, Boland A (2008). Training-specific changes in cardiac structure and function: a prospective and longitudinal assessment of competitive athletes. J Appl Physiol.

[2] Kim JH, Hollowed C, Liu C, Al-Badri A, Alkhoder A, Dommisse M (2019). Weight gain, hypertension, and the emergence of a maladaptive cardiovascular phenotype among US football players. JAMA Cardiol.

[3] Kim JH, Hollowed C, Patel K, Hosny K, Aida H, Gowani Z (2018). Temporal changes in cardiovascular remodeling associated with football participation. Med Sci Sports Exerc.

[4] Lin J, Wang F, Weiner RB, DeLuca JR, Wasfy MM, Berkstresser B (2016). Blood pressure and LV remodeling among American-style football players. JACC Cardiovasc Imaging.

[5] Weiner RB, Wang F, Isaacs SK, Malhotra R, Berkstresser B, Kim JH (2013). Blood pressure and left ventricular hypertrophy during American-style football participation. Circulation.

[6] Tso JV, Liu C, Turner CG, Uppal K, Prabakaran G, Ejaz K (2022). Metabolic alterations differentiating cardiovascular maladaptation from athletic training in American-style football athletes. Med Sci Sports Exerc.

[7] Tso JV, Turner CG, Liu C, Prabakaran G, Jackson M, Galante A (2023). Longitudinal aortic root dilatation in collegiate American-style football athletes. J Am Heart Assoc.

[8] Kim JH, Zafonte R, Pascuale-Leon A, Nadler LM, Weisskopf M, Speizer FE (2018). American-style football and cardiovascular health. J Am Heart Assoc..

[9] Zoghbi WA (2016). Cardiac remodeling in American-style football players: field position matters. JACC Cardiovasc Imaging.

[10] Weiner RB, Wang F, Berkstresser B, Kim J, Wang TJ, Lewis GD (2012). Regression of “gray zone” exercise-induced concentric left ventricular hypertrophy during prescribed detraining. J Am Coll Cardiol.

[11] Crouse SF, White S, Erwin JP, Meade TH, Martin SE, Oliver JM (2016). Echocardiographic and blood pressure characteristics of first-year collegiate American-style football players. Am J Cardiol.

[12] Tucker AM, Vogel RA, Lincoln AE, Dunn RE, Ahrensfield DC, Allen TW (2009). Prevalence of cardiovascular disease risk factors among National Football League players. JAMA.

[13] Baron SL, Hein MJ, Lehman E, Gersic CM (2012). Body mass index, playing position, race, and the cardiovascular mortality of retired professional football players. Am J Cardiol.

[14] Churchill TW, Krishnan S, Weisskopf M, Yates BA, Speizer FE, Kim JH (2018). Weight gain and health affliction among former National Football League players. Am J Med..

[15] Johri AM, Poirier P, Dorian P, Fournier A, Goodman JM, McKinney J (2019). Canadian Cardiovascular Society/Canadian Heart Rhythm Society joint position statement on the cardiovascular screening of competitive athletes. Can J Cardiol.

[16] Wellman AD, Coad SC, Flynn PJ, Siam TK, McLellan CP (2019). Comparison of preseason and in-season practice and game loads in National Collegiate Athletic Association Division I football players. J Strength Cond Res.

[17] Nguyen VT, Zafonte RD, Chen JT, Kponee-Shovein KZ, Paganoni S, Pascual-Leone A (2019). Mortality among professional American-style football players and professional American baseball players. JAMA Netw Open.

[18] Croft LB, Belanger A, Miller MA, Roberts A, Goldman ME (2008). Comparison of National Football League linemen versus nonlinemen of left ventricular mass and left atrial size. Am J Cardiol.

[19] Mosteller RD (1987). Simplified calculation of body-surface area. N Engl J Med.

[20] Lang RM, Badano LP, Mor-Avi V, Afilalo J, Armstrong A, Ernande L (2015). Recommendations for cardiac chamber quantification by echocardiography in adults: an update from the American Society of Echocardiography and the European Association of Cardiovascular Imaging. J Am Soc Echocardiogr.

[21] Goh VJ, Le T-T, Bryant J, Wong JI, Su B, Lee C-H (2017). Novel index of maladaptive myocardial remodeling in hypertension. Circ Cardiovasc Imaging..

[22] Greenberg NL, Vandervoort PM, Firstenberg MS, Garcia MJ, Thomas JD (2001). Estimation of diastolic intraventricular pressure gradients by Doppler M-mode echocardiography. Am J Physiol Heart Circ Physiol.

[23] Firstenberg MS, Smedira NG, Greenberg NL, Prior DL, McCarthy PM, Garcia MJ (2001). Relationship between early diastolic intraventricular pressure gradients, an index of elastic recoil, and improvements in systolic and diastolic function. Circulation.

[24] Hodzic A, Bonnefous O, Langet H, Hamiche W, Chaufourier L, Tournoux F (2020). Analysis of inter-system variability of systolic and diastolic intraventricular pressure gradients derived from color Doppler M-mode echocardiography. Sci Rep.

[25] Badano LP, Kolias TJ, Muraru D, Abraham TP, Aurigemma G, Edvardsen T (2018). Standardization of left atrial, right ventricular, and right atrial deformation imaging using two-dimensional speckle tracking echocardiography: a consensus document of the EACVI/ASE/Industry Task Force to standardize deformation imaging. Eur Heart J Cardiovasc Imaging.

[26] D’Ascenzi F, Pelliccia A, Natali BM, Cameli M, Andrei V, Incampo E (2015). Increased left atrial size is associated with reduced atrial stiffness and preserved reservoir function in athlete’s heart. Int J Cardiovasc Imaging.

[27] Hodzic A, Bernardino G, Legallois D, Gendron P, Langet H, De Craene M (2021). Right ventricular global and regional remodeling in American-style football athletes: a longitudinal 3D echocardiographic study. Appl Sci.

[28] Kim JH, Hollowed C, Irwin-Weyant M, Patel K, Hosny K, Aida H (2017). Sleep-disordered breathing and cardiovascular correlates in college football players. Am J Cardiol.

[29] Poirier P, Giles TD, Bray GA, Hong Y, Stern JS, Pi-Sunyer FX (2006). Obesity and cardiovascular disease: pathophysiology, evaluation, and effect of weight loss. Arterioscler Thromb Vasc Biol.

[30] de Morais N de S, Azevedo FM, de Freitas Rocha AR, Morais D de C, Ribeiro SAV, Gonçalves VSS, et al. Body fat is superior to body mass index in predicting cardiometabolic risk factors in adolescents. Int J Environ Res Public Health. 2023;20:2074. 10.3390/ijerph20032074 PMC9915438 36767439

[31] Le T-T, Lim V, Ibrahim R, Teo M-T, Bryant J, Ang B (2021). The remodelling index risk stratifies patients with hypertensive left ventricular hypertrophy. Eur Heart J Cardiovasc Imaging.

[32] Hodzic A, Garcia D, Saloux E, Ribeiro PAB, Ethier A, Thomas JD (2020). Echocardiographic evidence of left ventricular untwisting-filling interplay. Cardiovasc Ultrasound.

[33] Aeschbacher BC, Hutter D, Fuhrer J, Weidmann P, Delacrétaz E, Allemann Y (2001). Diastolic dysfunction precedes myocardial hypertrophy in the development of hypertension. Am J Hypertens.

[34] Wong CY, O’Moore-Sullivan T, Leano R, Byrne N, Beller E, Marwick TH (2004). Alterations of left ventricular myocardial characteristics associated with obesity. Circulation.

[35] Thomas L, Marwick TH, Popescu BA, Donal E, Badano LP (2019). Left atrial structure and function, and left ventricular diastolic dysfunction: JACC state-of-the-art review. J Am Coll Cardiol.

[36] Cuspidi C, Sala C, Tadic M, Baccanelli G, Gherbesi E, Grassi G (2019). Left atrial volume in elite athletes: a meta-analysis of echocardiographic studies. Scand J Med Sci Sports.

[37] McClean G, George K, Lord R, Utomi V, Jones N, Somauroo J (2015). Chronic adaptation of atrial structure and function in elite male athletes. Eur Heart J Cardiovasc Imaging.

[38] D’Ascenzi F, Anselmi F, Focardi M, Mondillo S (2018). Atrial enlargement in the athlete’s heart: assessment of atrial function may help distinguish adaptive from pathologic remodeling. J Am Soc Echocardiogr.

